# The ‘algebra of evolution’: the Robertson–Price identity and viability selection for body mass in a wild bird population

**DOI:** 10.1098/rstb.2019.0359

**Published:** 2020-03-09

**Authors:** G. K. Hajduk, C. A. Walling, A. Cockburn, L. E. B. Kruuk

**Affiliations:** 1 Institute of Evolutionary Biology, University of Edinburgh, Edinburgh EH9 3FL, UK; 2 Research School of Biology, Australian National University, Canberra, ACT 2601, Australia

**Keywords:** natural selection, quantitative genetics, Price equation, selection gradient, *Malurus*

## Abstract

By the Robertson–Price identity, the change in a quantitative trait owing to selection, is equal to the trait's covariance with relative fitness. In this study, we applied the identity to long-term data on superb fairy-wrens *Malurus cyaneus*, to estimate phenotypic and genetic change owing to juvenile viability selection. Mortality in the four-week period between fledging and independence was 40%, and heavier nestlings were more likely to survive, but why? There was additive genetic variance for both nestling mass and survival, and a positive phenotypic covariance between the traits, but no evidence of additive genetic covariance. Comparing standardized gradients, the phenotypic selection gradient was positive, *β*_P_ = 0.108 (0.036, 0.187 95% CI), whereas the genetic gradient was not different from zero, *β*_A_ = −0.025 (−0.19, 0.107 95% CI). This suggests that factors other than nestling mass were the cause of variation in survival. In particular, there were temporal correlations between mass and survival both within and between years. We suggest that use of the Price equation to describe cross-generational change in the wild may be challenging, but a more modest aim of estimating its first term, the Robertson–Price identity, to assess within-generation change can provide valuable insights into the processes shaping phenotypic diversity in natural populations.

This article is part of the theme issue ‘Fifty years of the Price equation’.

## 1. Introduction

Mathematical theory provides a useful framework with which to investigate the immense complexity of the biological world. In one of the best examples of its application, Price's theorem (or ‘the Price equation’; [1]) provides a simple and yet comprehensive means of describing the change in a biological entity across a chosen time-step. The Price equation constitutes the ‘algebra of evolution’ [2] and arguably deserves to be known as *the* ‘fundamental’ theorem of evolution [3], as it holds true in all situations, for all biological entities, and can be used to describe any biological dynamics at any scale, from population genetics to population ecology [3,4]. As outlined in detail in other papers in this Special Issue, the Price equation is based on the straightforward observation that the mean value of any entity in a population at a given time point is determined by two components: the frequency of different classes of individuals in the population, and the value of the entity within each of those classes. The *change* in the mean value of the entity across a given time-step is then determined by (i) the change in the relative frequency of the different classes and (ii) the change in value of the entity within each class across the time-step. This is such elegant and impeccable logic that it brings to mind Huxley's quoted response to Darwin's theory of natural selection: ‘How extremely stupid not to have thought of that!’ [5, p. 197].

The Price equation can be applied in a quantitative genetic framework, to describe the evolution of complex traits within populations. Thinking about the dynamics of a quantitative phenotypic trait, the change in the mean of the trait from one time point to another can be written as the sum of two components: the first owing to variation in fitness, which is the effect of selection changing the contributions from individuals of different trait values, and the second owing to changes in the expression of the trait over the time-step (which may be for various reasons, such as imperfect transmission or phenotypic plasticity) [1,2,6,7]. Although the Price equation is usually considered in the context of describing change *across* generations, it is also equally valid for considering change *within* a generation, for example owing to variation in survival—and even just to variation in survival of a particular life-stage, or episode of selection [2]. This application to a single episode of selection is obviously considerably simpler to analyse as there is no ‘transmission phase’. The Price equation then reduces to its first term, and simply asks: how does variation in survival of a particular episode of selection change the mean of a trait? Formally, the first term of the Price equation thus specifies the change in the mean of the trait, $\Delta \bar{z}$, owing to selection as

1.1 $$\Delta \bar{z}=S_{P}=\sigma_{P}(z,w),$$

where *S*_P_ is a selection differential and $\sigma_{P}(z,w)$ is the covariance between the phenotypic trait z  and relative fitness *w* [1] (we use a subscript P here to denote phenotypic values, to distinguish from what follows).

If there is heritable genetic variance for the trait, we can also consider whether the phenotypic selection will result in genetic change, which would then ultimately contribute to evolutionary change across generations. The corresponding change in the mean additive genetic value of the trait owing to variation in survival is an equivalent ‘genetic differential’ *S*_A_:

1.2 $$\Delta \bar{a}=S_{A}=\sigma (a_{z},w)=\sigma_{A}(z,w),$$

where *a*_z_ is the ‘breeding value’ (additive genetic merit) for *z*, and $\sigma_{A}$ is the additive genetic covariance between the trait and relative fitness [1,6]. Again, because we are only considering the contribution of selection to change *within* a generation, there is no transmission term to worry about. In a very nice example of the convergence of evolutionary theory, equation (1.2) was also derived at a similar time by a quantitative geneticist, Alan Robertson, in the context of predicting responses to selection in animal breeding [8,9]. As with the Price equation, there are interesting conceptual variations in the interpretation of Robertson's prediction (see [6], pp. 165 and p. 682 for an excellent discussion, including of the equivalence of the final two terms in equation (1.2)). However, for practical purposes, the key point is that the ‘Robertson–Price identity’ can be used to estimate within-generation change, at either the phenotypic level (equation (1.1) above) or the genetic level (equation (1.2)).

Although the Robertson–Price identity describes change within a generation, the ulterior motive for considering change in the genetic component of a trait is always likely to be because of the implications for change across generations, i.e. an evolutionary response*.* Thus the effect of selection in changing the mean of individuals' additive genetic components (or breeding values [10]) *within* a generation is equivalent to the contribution of that selection to changes in the mean phenotype *between* generations. The between-generational change in phenotypic mean owing to this selection, *R*_z_, is then also given by $R_{z}=\sigma_{A}(z,w)$; this is Robertson's Secondary Theorem of Natural Selection [9]. The genetic covariance between a trait and fitness is thus more typically considered in relation to predicting change from one generation to the next, because this is a larger question in which both evolutionary biologists and animal breeders are more likely to be interested. However, doing so is a much greater challenge for studies of natural systems experiencing natural selection on multivariate phenotypes and environmental heterogeneity, not least because it requires analysis of individuals' total lifetime fitness. Here, we consider only the contribution of a single episode of selection to phenotypic and genetic change, and hence the application of the Price equation to analysing within-generation change. Doing so is possible because of the convenient fact that the Price equation applies to any time-frame, and so does not require consideration of total lifetime fitness: we are not attempting a full description of observed cross-generational changes in phenotype. This is not a new point, but it is one that maybe often gets overlooked in the ambition to consider total evolutionary change. We show here that it is challenging enough to address the relatively modest aim of considering only within-generation change owing to an episode of viability selection of a single trait.

The process of estimating the additive genetic covariance can also provide useful insights into the operation of selection via a component of fitness [4,11]. We assume we are dealing with a heritable trait, i.e. one for which there is additive genetic variance, and we also assume (for now, but see below) that no other genetically correlated traits are relevant for fitness. If the trait *causes* variation in survival, the genes that shape the trait will also determine survival, and so breeding values for the trait will contribute to breeding values for survival. Alternatively, the trait might have no causal effect on survival, but if a different, exogenous variable (unrelated to individual genotypes) affects both trait and fitness, there will be a phenotypic covariance between the trait and fitness—but no genetic covariance, and no evolutionary relevance because the apparent selection cannot effect any genetic change [12–15]. Further, if there is a causal effect of the trait but also an effect of the endogenous variable, phenotypic covariances will be inflated (assuming the same direction of effects) or deflated (if in an opposing direction), relative to those owing just to the direct effect of the trait itself. Thus, comparison of phenotypic versus genetic associations between trait and fitness can give useful conceptual insights into whether a trait has a causal effect on fitness. In practice, any quantitative comparison requires standardization of the covariance parameters to the same scale, so the appropriate test is a comparison of the respective gradients:

1.3 $$\frac{\sigma_{P}(z,w)}{\sigma_{P}^{2}(z)}=\frac{\sigma_{A}(z,w)}{\sigma_{A}^{2}(z)}\;,$$

where the left-hand side is the phenotypic selection gradient *β*_P_ , the right-hand side is a ‘genetic gradient’ *β*_A_ [12,13,16,17] and *w* is the measure of relative fitness, or fitness component. (Note that *β*_A_ is sometimes referred to as *β*_G_, but we have used subscript A for consistency with the additive genetic (co)variance parameters.) The equivalence of the gradients *β*_P_ = *β*_A_ is, therefore, a test of the trait being the only cause of variation in fitness (or of variation in the focal component of fitness). An alternative arrangement of equation (1.3) is that the ratio of the two covariances $\sigma_{A}(z,w)/\sigma_{P}(z,w)$ equals the heritability of the trait, $\sigma_{A}^{2}(z)/\sigma_{P}^{2}(z)$. With multiple traits, if we relax the assumption that no other genetically correlated traits are relevant, and consider the vectors ***β*_P_** and ***β*_A_** of phenotypic and genetic gradients, the equality of ***β*_P_** = ***β*_A_** is a test of whether all genetically correlated traits affecting fitness are included in the analysis, i.e. of whether we have identified all causes of fitness variation [6,12,13].

If the trait *z* is the sole cause of variation in fitness and so equation (1.3) holds, the Secondary Theorem of Natural Selection combined with a re-arrangement of (1.3) shows that the cross-generational change in phenotypic mean *R*_z_ owing to the focal episode of selection is

1.4 $$R_{z}=\sigma_{A}(z,w)=\frac{\sigma_{A}^{2}(z)}{\sigma_{P}^{2}(z)}\sigma_{P}(z,w)=h^{2}S_{P}\;$$

This is the well-known ‘breeder's equation’ [18], whereby change in the mean of a trait is simply the product of the selection differential *S*_P_ and the trait's heritability *h*^2^. It provides an intuitively appealing way of describing a response to selection: the amount of change is determined first by how much selection changes the trait within a generation (*S*_P_), and then by how much of that change is transferred to the next generation (*h*^2^). Conceptually, the breeder's equation separates the ecology from the genetics [6, p. 687]. However, for the breeder's equation to accurately describe cross-generational change requires demanding assumptions. In the univariate formulation, it is assumed that there are causal effects of the focal trait on fitness (‘sole causality’ as stated above). If multiple traits are relevant, the assumption is that all genetically correlated traits with causal effects on fitness have been included in a multivariate formulation of equation (1.4) ([19], termed ‘joint-sole causality’ by [13]). Importantly, these assumptions may be feasible for artificial selection, when animal or plant breeders' decisions based on values of traits, or derived selection indices, are the ‘cause’ of variance in fitness—though, ironically, it is used less often in animal breeding than predictions based on indices [20]. However, the assumptions become much more tenuous when describing the effects of natural selection on multivariate phenotypes in natural populations, and unsurprisingly the breeder's equation does not perform well in natural populations [21,22]. By contrast, the prediction of the amount of genetic change owing to selection using the Robertson–Price identity, the additive genetic covariance, does not require such stringent assumptions. In addition, comparison of the phenotypic and selection gradients provides a test of sole causality of the focal trait(s) [12,13].

There is now increasing interest in applying these concepts to studies of natural populations. Although empirical analyses are challenging, additive genetic covariances between traits and fitness have now been estimated in a handful of wild vertebrates (for example: Soay sheep *Ovis aries* [23], bighorn sheep *Ovis canadensis* [24], red deer *Cervus elaphus* [25,26], snow voles *Chionomys nivalis* [27,28], great tits *Parus major* [29] and Atlantic salmon *Salmo salar* [30]), and also in evolutionary analyses of plant systems (e.g. morning glory *Ipomoea hederacea* [31]). Such analyses are also increasingly fitted with distributions that are appropriate for non-Gaussian fitness components [32,33]. It is also worth emphasizing that for a genetic covariance to occur obviously requires genetic variance in both the trait and fitness [13], and while heritability of many traits in wild populations is well established [34], there is still a surprising scarcity of estimates of additive genetic variance in fitness in wild populations [35].

To summarize, the Robertson–Price identity can be used to assess the potential for an episode of selection to cause phenotypic and genetic change in a trait within a generation, and also to shed light on whether a trait has a causal effect on a given component of fitness. This is arguably a more realistic ambition than a comprehensive description of the cross-generation dynamics of a trait. Here, we use this approach to investigate the relationship between body size and juvenile survival in a wild bird population. We analysed the role of a measure of total body size, mass, in causing variation in survival in a population of superb fairy-wrens (*Malurus cyaneus*) that has been the subject of a long-term study since 1988 [36,37]. Previous work on this population has shown that nestling mass is heritable and apparently under directional selection, with heavier mass being associated with a higher probability of early survival [38]. However, to date, there has been no evidence of any phenotypic change in mean nestling mass [39]. Superb fairy-wrens have exceptionally high levels of extra-pair paternity [38,40], with 85% of nests containing at least one extra-pair offspring; this mixed paternity of broods is statistically helpful for separating genetic from common environment sources of similarity between nest-mates. We fitted bivariate generalized ‘animal models’ to 26 years of data and a multigenerational pedigree up to 13 generations in order to separate the genetic and non-genetic components of the associations between mass and juvenile survival. The analysis allowed us to estimate the additive genetic covariance between nestling mass and survival, and to compare the phenotypic and genetic selection gradients, the equivalence of which would indicate that mass is the cause (specifically, the sole cause) of variation in survival.

## 2. Material and methods

### (a) Study system

The study population of superb fairy-wrens is located in an approximately 60 ha area in and around the Australian National Botanic Gardens, Canberra, Australia (35°16 S, 149°06 E). All individuals in the population are colour-banded, and the population is censused throughout the year at weekly intervals (if a bird is not sighted on routine censuses, deliberate attempts are made to find it, so sighting probabilities exceed 99%) [36]. Superb fairy-wrens are cooperative breeders: breeding pairs may be assisted by up to four (in one exceptional case, five) male helpers, who help the parents in provisioning the offspring [41]. They are also multi-brooded, with a long breeding season that extends from September to March of the following year [42]. Owing to heavy nest predation, a female may initiate up to eight clutches in a given year, but will only ever raise a maximum of three broods to fledging each year. Clutches contain one to five eggs, with a strong mode at three eggs [43].

During the breeding season, the progress of all nests is monitored, with nestlings weighed and banded 5–8 days post-hatching, and date of fledging and subsequent fate of fledglings closely monitored. Censuses at this time of year are conducted at least three times each week [36], so death dates for each individual can be estimated accurately. Here, we considered survival from fledging (typically at age 13 days) to independence, defined as surviving at least four weeks after fledging (or to age 41 days since hatching). Since we were interested in individual-level associations of phenotype with survival, this post-fledging survival—i.e. after leaving the nest—is more relevant than survival in the nest, when mortality is almost entirely owing to predation of the entire brood. This period is also after the date on which nestlings will have been banded and weighed. We used an upper bound of four weeks post fledging as the youngest possible age of independence. Most young are still being provisioned at this age, but the earliest known dispersal in our study happened at four weeks post fledging: this cut-off point, therefore, avoids any chance of dispersal being confused with mortality. Blood samples were taken from nestlings at banding, and used for parentage assignment using microsatellite genotypes (see methods in [38]). From this parentage assignment, we constructed a multigenerational pedigree with a maximum lineage length of 13 generations. Summary statistics for the traits and pedigree are given in electronic supplementary material, table S1, but in brief: we used data from 26 breeding seasons from 1988 to 2013, for a total of 3808 nestlings from 1472 nests.

### (b) Statistical analyses

We fitted a bivariate generalized linear mixed model using a Bayesian framework implemented in the *R* package *MCMCglmm* [32], with response variables of nestling mass and survival from fledging to independence. We used an animal model approach [44] incorporating the pedigree information to partition (co)variances into additive genetic and several non-genetic components. For both traits, we fitted a fixed effect of *nestling sex* (known because all nestlings were genotyped using the CHD test [45]) and several other fixed effects to account for other variables that may affect either mass or survival: the *number of helpers* in the group (as a three-level factor: 0, 1 and 2+; where the ‘2+’ level consisted mainly of 2 helpers [39]; *brood size* (the number of nestlings in a given brood, as a covariate ranging from 1 to 5), to account for potential variation in the amount of care provided to individual nestlings; and the pedigree *inbreeding coefficient* of each individual, to account for inbreeding depression [38]. We also tested for differences in performance between offspring of *extra-pair* versus *within-pair* copulations (following [46]), by fitting an additional two-level factor of whether or not the nestling was the offspring of the dominant male on the territory. In addition, for nestling mass, we fitted *nestling age* in days, ranging from 5 to 8, and fitted as a covariate with a quadratic function to represent growth over the nestling period. Finally, as in previous analyses of nestling mass [38,39], we fitted a two-level factor to correct for a change in field protocol: prior to 1992, chicks were weighed throughout the day and so were on average heavier than in later cohorts, which were always weighed in the early morning (‘*Pre1992*’ factor).

The model contained random effects of an *additive genetic effect*, with covariance structure determined by the pedigree, to estimate the additive genetic (co)variances [44], and *nest identity* to account for covariance owing to the common environments shared by offspring in the same brood [47]. We also modelled two forms of temporal variation: inter-annual variation with a 26-level factor of *cohort* (1988–2013: the ‘1988’ cohort incorporates nestlings from September 1988 to March 1989, etc.) and intra-annual variation within a breeding season with a multi-level factor of *hatch-date* fortnight (split into 12 two-week intervals, between 23 September and 15 March). We explored fitting a maternal effect, specified by the identity of the mother, to test for consistent effects—both genetic and non-genetic—of individual mothers across all nests they produced. However, in univariate models of each trait (with the same random effects as specified above), there was no evidence of any maternal effect, nor of any change in the estimate of additive genetic variance when including a maternal effect (electronic supplementary material, table S2), indicating that common environment effects are driven by differences between individual nests more than between individual mothers. We also had trouble with bivariate models including a maternal effect converging, and therefore did not include a maternal effect in the bivariate models presented here.

The main bivariate model estimated components of variance and covariance between mass and survival for the random effects of nest, cohort, fortnight and additive genetic effects. For each sample of the posterior distribution, we estimated the corresponding correlation between traits for each random effect by dividing the covariance term by the square root of the product of the respective variances: this gave, for example, the correlation between cohort effects on each trait. Similarly, we estimated the total phenotypic variance for each trait (after correcting for fixed effects) as the sum of all the variance components, and the total phenotypic covariance as the sum of all covariances. Finally, we estimated total ‘environmental’ (i.e. non-genetic) variances and covariances, defined as the respective phenotypic (co)variances minus the additive genetic (co)variances.

All random effects were fitted for both response traits using ‘unstructured’ (*us*) covariance matrices, and the residual variance was set using *rcov* with an unstructured covariance matrix. Models were run for 5.2 × 10^6^ iterations, with a burn-in of 1.2 × 10^6^, thinning interval of 2000 and parameter expanded priors; code for the *MCMCglmm* model is provided in the electronic supplementary material. The effective sample sizes for specific parameters varied owing to auto-correlation, but we ensured that they were always above 1000. For each parameter, we report means of the posterior distribution and 95% credible intervals (CIs), which are defined as the shortest interval of the posterior distribution that contained 95% of the distribution. We considered there to be statistical support for a fixed effect or covariance of random effects if the 95% CIs did not span 0 and, for fixed effects, if pMCMC (the proportion of the posterior distribution that was smaller than 0) was <0.05.

This main model (model I) fitted nestling mass with Gaussian errors, whereas survival was treated as a binary variable fitted with *MCMCglmm*'s ‘threshold’ distribution, a probit link function and residual variance was fixed to 1 following standard MCMCglmm convention [32]; note that although the residual variance is fixed to 1, the residual covariance with nestling mass is still estimable. All parameter estimates for survival, including the variance and covariance parameters, are, therefore, on the probit latent scale. While this formulation is necessary for a model with an appropriate statistical distribution, it has implications for interpretation of the estimates of the covariance components, as the resulting parameters involve latent-scale fitness and so are not directly comparable to typical selection differentials or gradients. In the case of a fitness component with a log-normal distribution (which might, for example, be modelled with a Poisson distribution with log link function), there is a very convenient equivalence of estimates of (co)variance of *absolute* fitness on the latent-scale with the value of (co)variance of *relative* fitness on the data-scale [48,49], and so estimates from a log-normal GLMM can be used as standardized coefficients. Unfortunately, this correspondence does not hold for either probit or logit link functions (M. Morrissey 2019, personal communication). To generate the regression coefficients required to estimate the gradients considered in equation (1.3) above, we therefore fitted a second bivariate animal model (model II) of standardized nestling mass and relative survival, with both variables modelled with Gaussian error distributions. The covariances from this model estimate the linear differentials required for equation (1.3): note that although the statistical model does not use the appropriate error distribution for survival, the linear differential is the appropriate parameter that quantifies the change in the mean of the trait owing to selection [2,50].

In order to generate standardized selection gradients in model II, each individual's relative survival was calculated by dividing its observed survival (0 or 1) by the mean survival rate in its cohort, so that every cohort would have a mean relative survival of 1. We also standardized nestling mass to unit variance so that the resulting parameters in model II were standardized selection coefficients (note that in model I, mass is not standardized). For each sample of the posterior distribution for model II, we calculated the phenotypic selection gradient *β*_P_ (the left-hand side of equation (1.3)) by dividing the total phenotypic covariance by the total phenotypic variance in mass (defined as above). We also calculated the ‘genetic gradient’ *β*_A_ defined as the additive genetic covariance divided by the genetic variance, and finally, for completeness, the ‘environmental’ gradient *β*_E_, where the environmental (co)variance was the sum of all (co)variances except the additive genetic (see also [25,31]). This gave posterior distributions of the estimates of the three gradients, *β*_P_ , *β*_A_ and *β*_E_.

As a final point, inclusion of the fixed effects made little difference to the estimates of the (co)variance components in which we were primarily interested, but for comparison [51] we also present in the electronic supplementary material, table S3 a version of model I with the same random effects as above, but with only the essential fixed effects of sex, age and field-protocol-change. We also estimated selection gradients from the equivalent version of model II (i.e. without additional fixed effects), which again were very similar to those from the model with all fixed effects (electronic supplementary material, table S3 legend).

## 3. Results

**Figure 1.**
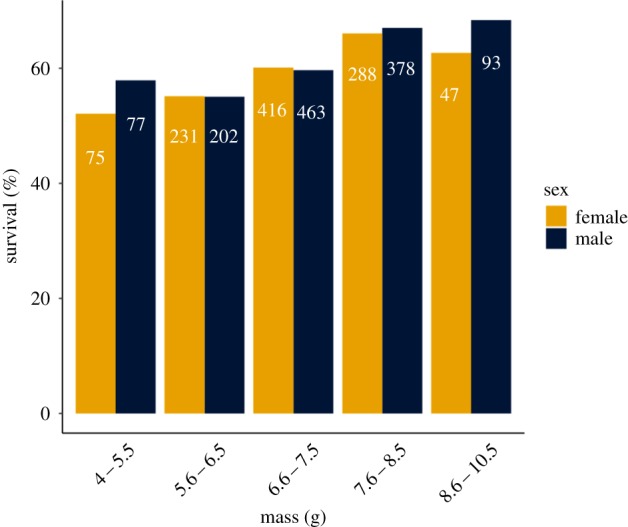
The relationship between nestling mass (binned into approximately 1 g categories) and survival from fledging to independence (12–41 days). The sample sizes of individuals in each group are given within the bars. (Online version in colour.)

Overall, 60% of female and 61% of male superb fairy-wren juveniles survived the four-week period from fledging to independence. Rates of survival increased with mass in both sexes (figure 1). In the bivariate model I of nestling mass and survival, there was a positive total phenotypic covariance between the two traits of Cov_P_ = 0.14 (0.05, 0.26 95% CI). Nestling mass also increased with age at measurement and with more helpers at the nest, and was higher for males relative to females, whereas it decreased with increasing brood size and with higher inbreeding coefficient. Survival was higher in nests with two or more helpers (table 1).

**Table 1.** Model of the components of variance and covariance between nestling mass and survival from fledging to independence. The fixed effects were nestling sex, age at measurement (in days, fitted as a quadratic), the effect of the change in weighing protocol in 1992, brood size, the number of helpers in the group, the nestling's inbreeding coefficient, and whether or not it was the result of extra-pair paternity (EP, versus within-pair WP). The model was fitted in MCMCglmm with a Gaussian distribution for nestling mass and a threshold distribution for survival. The model estimates are based on posterior means, 95% credible intervals (CIs) are given in brackets, and *p*-values are based on pMCMC. Parameter estimates for survival terms are on the logit scale. The analysis is of 3808 nestlings in 1472 nests across 26 years (see electronic supplementary material, table S1 for details of sample sizes). Italics indicate pMCMC < 0.05.

	nestling mass		survival from fledging to independence	
fixed effects	estimate (95% CI)	*p*	estimate (95% CI)	*p*
intercept	−3.35 (−6.00, −0.94)	*0.005*	0.77 (0.15, 1.38)	*0.013*
1992 (1992+, pre-1992)				
pre-1992	0.53 (0.33, 0.72)	*<0.001*		
nestling age	2.06 (1.37, 2.81)	*<0.001*		
nestling age^2^	−0.08 (−0.13, −0.03)	*<0.001*		
sex (female, male)				
male	0.15 (0.11, 0.19)	*<0.001*	0.04 (−0.10, 0.16)	0.574
brood size	−0.05 (−0.09, −0.01)	*0.022*	−0.10 (−0.23, 0.04)	0.138
helpers (0, 1, 2+)				
1 helper	0.08 (0.01, 0.15)	*0.050*	0.19 (−0.04, 0.42)	0.109
2+ helpers	0.15 (0.06, 0.24)	*0.002*	0.27 (0.01, 0.53)	*0.037*
inbreeding coefficient	−3.23 (−5.98, −0.43)	*0.028*	−4.04 (−13.60, 5.48)	0.423
within-pair status (EP, WP)				
WP	0.004 (−0.04, 0.05)	0.855	0.09 (−0.05, 0.24)	0.222

random effects	variance–covariance–correlation matrices (95% CI)			
	mass		survival	
nest ID				
mass	0.23 (0.20, 0.25)		0.05 (−0.04, 0.15)	
survival	0.03 (−0.03, 0.09)		1.60 (1.12, 2.02)	
hatch-date				
mass	0.01 (0.002. 0.03)		0.70 (0.23, 1.00)	
survival	0.07 (−0.01, 0.15)		0.71 (0.17, 1.54)	
cohort				
mass	0.01 (1.06^−5^, 0.02)		0.56 (0.03, 0.96)	
survival	0.02 (−0.001, 0.05)		0.15 (0.04, 0.31)	
additive genetic effect				
mass	0.09 (0.06, 0.13)		−0.09 (−0.53, 0.28)	
survival	−0.02 (−0.09,0.05)		0.39 (0.07, 0.84)	
residual variance				
mass	0.19 (0.17, 0.21)		0.11 (−0.01, 0.22)	
survival	0.05 (−0.003,0.10)		fixed to 1.00	
sample size	3808			

There was additive genetic variance for both nestling mass (*V*_A_ = 0.09 (0.06, 0.13 CI) and survival (*V*_A_ = 0.39 (0.07, 0.84 CI); table 1), explaining 17% (11, 24% CI) of the variance in mass (i.e. the heritability) and 10% (3, 18% CI) of the (latent-scale) variance in survival (electronic supplementary material, figure S1). There was also considerable variance between nests, accounting for 43% (39, 47% CI) of the variance in mass and 42% (32, 51% CI) of the variance in survival (table 1). Hatch-date contributed 2% (0.3, 5% CI) of the variance in nestling mass and 18% (6, 34% CI) of the variance in survival, whereas cohort contributed 2% (0, 3% CI) of the variance in mass and 4% (1, 8% CI) of the variance in survival.

**Figure 2.**
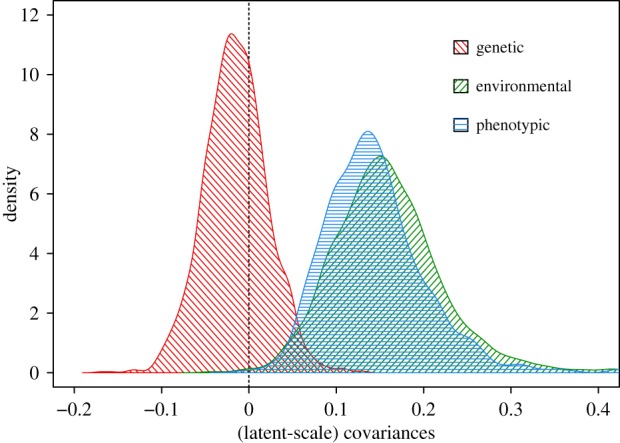
Posterior distributions of the estimates of components of covariance between nestling mass and juvenile survival in superb fairy-wrens (from model I, table 1). Red diagonal hashed lines indicate the additive genetic covariance, green diagonal lines indicate the environmental (non-genetic) covariance (defined as in §2) and blue horizontal lines indicate the phenotypic covariance. Survival was modelled as a threshold trait with a probit link function, so the parameter estimates are on the latent scale. Despite the positive phenotypic covariance and additive genetic variance for both traits (electronic supplementary material, figure S1), there was little support for positive additive genetic covariance.

The posterior means for the covariances and correlations of the different random effects were all positive, with the exception of those between the additive genetic effects (table 1). The additive genetic covariance was slightly negative, but with large CIs (Cov_A_ = −0.02 (−0.09, 0.05 CI); figure 2).

The total environmental covariance between mass and survival was positive (Cov_E_ = 0.16 (0.03, 0.28 CI); figure 2). For both nest and residual effects, the covariances were positive, but with CIs that overlapped zero. The associations between the two temporal terms, cohort and hatch-date, were also both positive, but their statistical support was complex. For the covariances, credible intervals for both terms just overlapped zero (table 1), but only 1.3% of the posterior distribution for hatch-date covariance and 3.4% of the cohort covariance was negative. The temporal terms had the strongest correlations: cohort correlation = 0.56 (0.03, 0.96 CI), hatch-date correlation = 0.70 (0.23, 1.00 CI; table 1), and their CIs did not overlap zero. We consider the slight mismatch between the level of statistical support for the covariances versus the correlations in §4.

**Figure 3.**
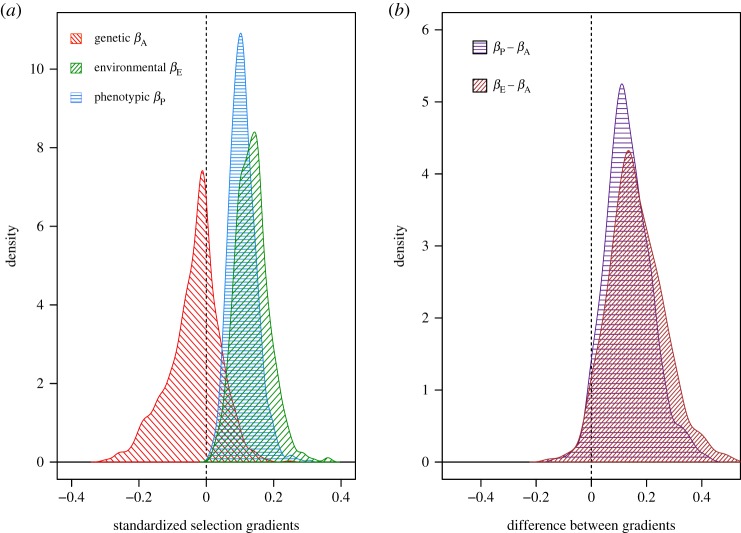
Posterior distributions of the estimates of the selection gradients for nestling mass. (*a*) Genetic and non-genetic gradients: red diagonal hashed lines indicate the additive genetic gradient *β*_A_, green diagonal lines indicate the environmental (non-genetic) gradient *β*_E_ and blue horizontal lines indicate the phenotypic gradient *β*_P_. (*b*) Tests of equivalence of gradients. Purple horizontal lines indicate the difference $\left(\beta_{P}-\beta_{A}\right)$ between the phenotypic and genetic gradients, and brown diagonal lines indicate the difference $\left(\beta_{E}-\beta_{A}\right)$ between environmental and genetic gradients. (Online version in colour.)

We then used a bivariate model with standardized mass and relative survival fitted as a Gaussian variable (model II) to estimate standardized selection gradients and test for the consistency of the phenotypic selection and genetic gradients. The standardized phenotypic selection gradient was *β*_P_ = 0.108 (0.036, 0.187 CI), whereas the genetic gradient was *β*_A_ = −0.025 (−0.19, 0.107 CI). The uncertainty on *β*_A_ was, therefore, sufficient that we cannot confidently conclude that the phenotypic and genetic gradients (from equation (1.3)) were different, but we note that each posterior mean falls outside the CI of the other parameter, especially the posterior mean of *β*_A_ (figure 3*a*). The difference between the two estimates $(\beta_{P}-\beta_{A}),$ estimated for each sample of the posterior distribution, had a posterior mean of 0.133 (−0.017, 0.316 CI; figure 3*b*), and *β*_P_ was greater than *β*_A_ in 96.1% of the samples. As a final test (following [17]), we compared the genetic gradient *β*_A_ with the environmental gradient *β*_E_, which had posterior mean 0.137 (0.050, 0.239 CI). The mean difference $\left(\beta_{E}-\beta_{A}\right)$ was 0.161 (−0.030, 0.364) and was also positive for 96.1% of its distribution. Overall, while there was clear evidence of positive phenotypic and environmental gradients, in contrast to the slightly negative genetic gradient, the uncertainty on the parameters was sufficient that CIs for the differences still just overlapped zero (figure 3).

## 4. Discussion

This paper is part of a Special Issue on the Price equation. Using a quantitative genetic approach, we have focussed on the first term of the Price equation, the Robertson–Price identity, and its application to describing the effect of selection in instigating genetic change. Estimation of this covariance provides a useful test of the impact of selection on a trait. It is highly unlikely that either of the two authors of the Robertson–Price identity, Alan Robertson and George Price, would ever have imagined that their quantitative genetic ‘algebra of evolution’ [2] would one day be applied to data from a small Australian songbird known for its bright blue plumage and high levels of infidelity. However, we hope to have shown here how the process of estimating the Robertson–Price covariance can provide both evolutionary and ecological insights into the dynamics of a natural system. Below, we first consider the broader evolutionary issues that arise from the quantitative genetic analyses, and then discuss the results concerning sources of variation in morphology and fitness in the superb fairy-wren study population.

The focal phenotypic trait here, nestling mass, constitutes yet another example of a heritable trait apparently under positive directional selection, for which the breeder's equation would therefore predict a cross-generation response to selection [18]. However, there has been no indication of any phenotypic change in mean nestling mass across the study period [39]. This could be for many reasons, such as confounding effects of phenotypic plasticity in response to any environmental change, or countering selection acting via other components of fitness [21]. However, comparison of the phenotypic versus genetic gradients for survival on mass provides a test of the breeder's equation assumption that all traits with causal effects on fitness are incorporated in our model. Our estimates of the additive genetic covariance and the corresponding genetic gradient have large CIs, but overall there is little support for the breeder's equation's assumption of the equality of phenotypic versus genetic gradients (figure 3). Under this assumption, breeding values for mass would contribute to breeding values for survival, generating a genetic covariance between the two [13], which we do not observe.

**Figure 4.**
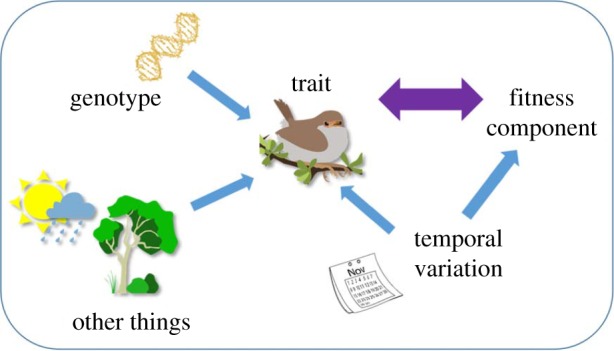
A phenotypic trait in an individual may be determined by a range of factors, including the individual's genotype and environmental variation; these effects are depicted by single-headed arrows. The diagram represents a scenario whereby both the trait of interest (such as body size) and a component of fitness change in a correlated manner owing to temporal variation (both within or between years), generating a statistical correlation between trait and fitness depicted by the double-headed purple arrow. The net result is a phenotypic covariance between trait and fitness, and hence the appearance of selection, but no potential for any evolutionary response. After [14,15], but considering, in particular, the confounding effects of temporal variation. (Online version in colour.)

Rather than a causal relationship between mass and survival, our results suggest that other confounding sources of variance are generating covariance between mass and survival in this population. In particular, temporal variation may be contributing to the phenotypic association: the strongest correlations between mass and survival were those owing to the effects of differences between hatch-date intervals and cohorts. There was also weak support for a positive correlation across nests, suggesting that nests with heavier nestlings had higher survival rates after leaving the nest (table 1). This may, therefore, be a situation in which confounding effects of extrinsic variables result in an overall phenotypic covariance between a trait and fitness, but there is no potential for any evolutionary response because the covariance lacks a genetic component (figure 4). Temporal variation would thus be a particular case of external extrinsic conditions generating the appearance of selection that can have no evolutionary relevance [12,15,52].

We considered here the effects of a single episode of selection, acting via differential survival over a period of time in early life. This is a short period, but it is important, as average survival was only 60–61%: the covariance of relative survival of this period with relative lifetime breeding success was 0.56 (where lifetime breeding success is the total number of offspring produced, which will be zero for those that died as juveniles). We have estimated just the first term of the full Price equation, the additive genetic covariance (or Robertson–Price identity), but because we are considering the change in an entity (genetic breeding value for nestling mass prior to 9 days) that cannot change during the period of selection (age 13–41 days), our implementation is effectively of the full Price equation for the change caused by this episode of selection. Put differently, we are not making assumptions about the second term of the Price equation being negligible, but rather we are only considering a time interval for which, by definition, the second term is zero. As such, the application of the Price equation to describe within-generation change is arguably where it can most realistically be used for data from natural populations. We are not aiming for comprehensive representation of the likely change in genetic (let alone phenotypic) values from one generation to the next—but rather for a measure of how much change there will have been owing to one episode of selection that would ultimately contribute to cross-generation change. Fairy-wrens will face numerous other selection pressures in their lives that will shape overall variation in fitness. Nevertheless, body size is arguably the most well-studied trait as a target of selection (for example, constituting 20% of more than 5000 estimates of selection reviewed in [53]), and juvenile survival possibly the most easily measured component of fitness, so the example may be relevant to other studies. It may also be that in other species and populations there are clearer reasons for causality to underlie any correlation between mass and survival. For example, in many other wild animal populations (if not here), starvation may be the most likely cause of juvenile mortality, and so assumptions of causal associations between size and survival may be more plausible. Thus the extent to which our results hold across other systems will be interesting to see. It is also possible that changing climates alter selection on morphology in wild populations—although, notably, two recent meta-analyses have not found evidence of selection coefficients on body size changing with either temperature or time [54,55].

On the statistical front, our study illustrates several points that will be relevant for similar analyses. First, we modelled the binary trait of survival (survived or did not survive, yes/no) as a threshold distribution, with a link function of a probit transformation [56]. The covariance estimated in the model is, therefore, the covariance between the data-scale nestling mass values and the latent-scale survival, and so is not readily interpretable as a selection differential in the standard sense. Similar GLMMs that use a log-normal model (such as for a Poisson distribution) have the useful characteristic that latent-scale variances and covariances of absolute fitness are equivalent to data-scale variances and covariances with relative fitness [48]. Unfortunately, this correspondence does not apply for models using probit or logit links. We therefore estimated selection differentials in separate models assuming Gaussian errors with relative fitness and an identity link function. An alternative approach would be the transformation of the GLMM parameters, for example using QGglmm [33], to derive data-scale estimates of the covariances with survival, which could then be transformed to give covariances with relative survival. Second, on a subtle point regarding MCMCglmm output, the 95% CIs for the covariances for the temporal components just overlapped zero, but those for the correlations did not (table 1). We do not want to put too much weight on an apparent marginal statistical significance of one parameter and not another (see e.g. arguments in [57]), but this potentially puzzling contrast is probably the result of the exact nature of MCMCglmm's CIs, which are the shortest interval that contains 95% of the posterior distribution [32]. If distributions are asymmetric, the CI will favour the ‘fatter’-tailed end of the distribution, so the difference here probably reflects a difference in the directions of skew of the posterior distributions of the covariance versus the correlation statistics (shown in electronic supplementary material, figure S2).

Finally, whilst we very much want to encourage other similar analyses, it is worth noting that MCMC analyses such as those presented here are computationally demanding. Our main bivariate model took three weeks to run on a central mainframe computer, for a dataset that is large but by no means unusual for a long-term study; it fell over completely when we tried to incorporate a sixth random effect. Whether there is sufficient statistical power for reasonable estimates of genetic covariances remains a perennial problem for studies of wild populations [58], and our conclusions here are obviously constrained by the substantial uncertainty on the estimates of additive genetic covariance and genetic gradient. Nevertheless, although estimates from other wild populations are scarce [23,25–27,29], there has been clear support for non-zero additive genetic covariance in at least two other cases (body size in snow voles [27] and breeding time in red deer [26]), so its detection in the wild is possible.

Our analyses also described other factors relevant to early life performance in juvenile superb fairy-wrens, with the results matching previous studies on this population showing effects of age, sex and social environment (table 1; [38,39]). We also tested for differences in either mass or survival between within-pair versus extra-pair offspring, but found no effect (table 1). However, despite the statistical support for the majority of the fixed effects included in the model, they did not account for a large amount of the total variance: an equivalent model to that in table 1 but with only the ‘baseline’ fixed effects of sex, age and protocol had very similar variance and covariance components of the random effects (electronic supplementary material, table S3). There was however substantial variance between nests for both mass and survival (table 1), presumably reflecting unmeasured characteristics of the territory and food resources, or the nest itself (its location, how inconspicuous it is, etc.). The variance between nests may also reflect variation associated with the mother, the dominant male on the territory and the identity of the helpers attending the nest, although there was little evidence of consistent differences between mothers in their effects across breeding seasons and years (i.e. of consistent ‘maternal effects’, electronic supplementary material, table S2).

A lack of causality underlying the association between fairy-wren nestling mass and survival in the post-fledging period makes biological sense given that the most likely cause of mortality at this stage is predation of young fledglings, and it is not obvious why this should vary with mass. It is more plausible that predation and mass both vary between different times of the breeding season and between different years: times of higher predation may coincide with times of lower availability of fairy-wren food and hence lower mass. Variance owing to hatch-date (modelled here as the different fortnights through the breeding season) explained 18% of the variance in survival to independence, presumably owing to the effects of changing ecological conditions through the long fairy-wren breeding season. In particular, predation rates are likely to be highest during periods in which the predators themselves are raising young. Many of the nests in the study population are raided by pied currawongs (*Strepera graculina*), which will take nestlings and juveniles of other bird species to feed their young [59]; this predation may be highest during the earlier stages of the fairy-wren breeding season, which may be when abundance of the invertebrates on which superb fairy-wrens feed is lower. More detailed exploration of the causes of this temporal covariation will therefore be interesting, including extension of the analyses to model auto-correlation between adjacent years or hatching periods.

## 5. Conclusion

Quantitative genetic analyses in the form of multivariate animal models can be used to estimate the additive genetic covariance and other potential sources of covariance between traits and components of fitness. In particular, estimation of the Robertson–Price identity provides a test of whether a particular episode of selection will cause genetic change in a trait, and comparison of the corresponding phenotypic and genetic gradients indicates whether the focal trait is the sole cause of variation in fitness. In our superb fairy-wren study population, nestling mass was heritable and heavier individuals had a higher probability of survival, but the estimation of the Robertson–Price identity indicated that the selection would not generate any change in mean breeding value in the population. The positive environmental covariance between mass and survival was probably owing, at least in part, to correlated inter- and intra-annual temporal variation. In studies of wild populations, application of the Price equation—or even part of it—and tests of the equivalence of selection gradients may be challenging, but the process can offer useful insights into the causes and consequences of key episodes of natural selection in wild populations.

## Supplementary Material

Supplementary material: Selection on fairy-wren body mass

## Data accessibility

Statistical code and supporting data are provided at https://dx.doi.org/10.5061/dryad.3ffbg79dq.
