# Supplementary material for: The ‘algebra of evolution’: the Robertson–Price identity and viability selection for body mass in a wild bird population
Source: Philos Trans R Soc Lond B Biol Sci. 2020 Mar 9;375(1797):20190359. doi: 10.1098/rstb.2019.0359 (PMC7133512; doi:10.1098/rstb.2019.0359)
Supplement: Supplementary material: Selection on fairy-wren body mass [file rstb20190359supp1.docx]

**SUPPLEMENTARY INFORMATION**

**The ‘algebra of evolution’: the Robertson-Price covariance and viability selection for body mass in a wild bird population**

**Hajduk, G.K, Walling, C.A., Cockburn, A. and Kruuk, L.E.B.**

Contribution to Philosophical Transactions Royal Society B Special Issue on the Price Equation

## Supplementary Information contents

*Table S1:* Summary statistics.

*Table S3:* Univariate animal models with and without maternal effects.

*Table S2*: Bivariate animal model between nestling mass and juvenile survival (model I) without additional social environment fixed effects.

*Figure S1*. Posterior distributions of phenotypic, genetic and non-genetic components of variance in nestling mass and survival.

*Figure S2*. Posterior distributions of covariances and correlations between cohort and hatch-date random effects.

## Data accessibility

Statistical code and supporting data are provided at https://doi.org/10.5061/dryad.3ffbg79dq.

## Table S1. Summary statistics.

*Sample sizes and summary statistics for nestling mass and for post-fledging survival (ages 13-41 days, or 4 weeks post fledging), and for the overall pedigree, for years 1988-2013. Note that the ‘Total’ number of individuals is slightly larger than the sample size for each trait alone because 51 birds had records of mass but not survival, and 15 birds had records of survival but not mass; the same applies to the number of nests. The pedigree summary statistics are from the PedStatSummary in the R package pedantics [1].*

|  | N individuals | N nests | Mean | SD |
| --- | --- | --- | --- | --- |
| Nestling mass (g) | 3793 | 1471 | 7.076 | 0.978 |
| Survival (fledging to independence) | 3757 | 1471 | 0.607 | 0.488 |
| Total | 3808 | 1472 |  |  |
| Pedigree Statistics | |  |  |  |
| Total N individuals pedigree | 5982 |  |  |  |
| maternities | 5333 |  |  |  |
| paternities | 5252 |  |  |  |
| full sibs | 9704 |  |  |  |
| maternal siblings | 39595 |  |  |  |
| maternal half-siblings | 29891 |  |  |  |
| paternal sibs | 40584 |  |  |  |
| paternal half sibs | 30880 |  |  |  |
| maternal grandmothers | 1630 |  |  |  |
| maternal grandfathers | 1350 |  |  |  |
| paternal grandmothers | 4094 |  |  |  |
| paternal grandfathers | 3662 |  |  |  |
| maximum pedigree depth | 13 |  |  |  |
| founders | 620 |  |  |  |
| mean maternal sibsip size | 9 |  |  |  |
| mean paternal sibsip size | 7 |  |  |  |

## Table S2. Models with maternal effects.

*Univariate models for both mass and survival, fitted without and then with a maternal effect (MotherID), in addition to the other random effects of Nest, Cohort, Fortnight, and Additive Genetic Variance (V_A_). There was no support for a maternal effect in either trait, nor any evidence that its inclusion changed the estimates of other variance components (in particular, that of V_A_, which is shaded grey). We therefore did not include a maternal effect in the main bivariate models.*

|  | Variance components (and 95% CIs) | | |  |
| --- | --- | --- | --- | --- |
|  | Mass |  | Survival |  |
|  | *No Maternal Effect* | *With Maternal Effect* | *No Maternal Effect* | *With Maternal Effect* |
| Nest | 0.227 (0.200, 0.253) | 0.223 (0.197, 0.253) | 1.658 (1.230, 2.189) | 1.609 (1.170, 2.173) |
| Cohort | 0.007 (0.000, 0.017) | 0.007 (0.000, 0.016) | 0.164 (0.034, 0.318) | 0.157 (0.032, 0.322) |
| Fortnight | 0.013 (0.001, 0.032) | 0.012 (0.001, 0.030) | 0.785 (0.166, 1.717) | 0.764 (0.134, 1.753) |
| V_A_ | 0.093 (0.059, 0.131) | 0.089 (0.057, 0.130) | 0.468 (0.098, 0.948) | 0.438 (0.011, 0.908) |
| MotherID |  | 0.007 (0.000, 0.021) |  | 0.041 (0.000, 0.141) |
| Residual | 0.186 (0.161, 0.209) | 0.188 (0.164, 0.208) | 1.00 | 1.00 |

## Table S3. Bivariate model without fixed effects.

Model of the components of variance and covariance between nestling mass and survival from ﬂedging to independence, but excluding all but the essential fixed effects of nestling sex, age at measurement (in days, fitted as a quadratic), and the effect of the change in weighing protocol in 1992 (as in Table 1). The model estimates shown are posterior means with 95% credible intervals (CIs) are given in brackets, and p-values for fixed effects based on pMCMC. Please note that parameter estimates for survival terms are on the logit scale. The analysis is of 3808 nestlings in 1472 nests across 26 years (see Table S1). Estimates of standardised selection gradients from an equivalent version of model II (i.e. with only the essential fixed effects as here) were $\beta_{P}$= 0.108 (0.033, 0.184 CI) and $\beta_{A}$= 0.024 (0.216, 0.104 CI).


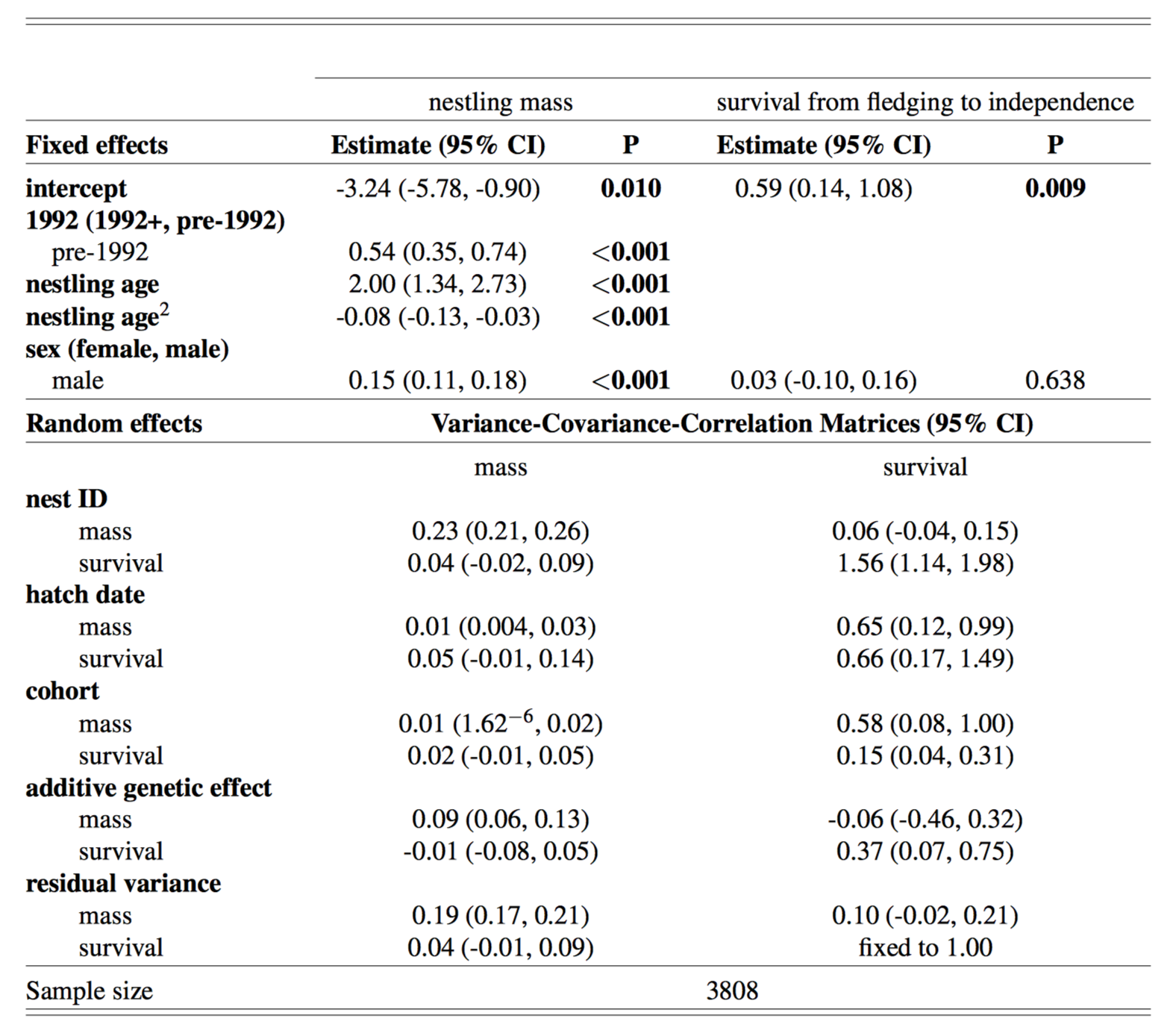


## Figure S1


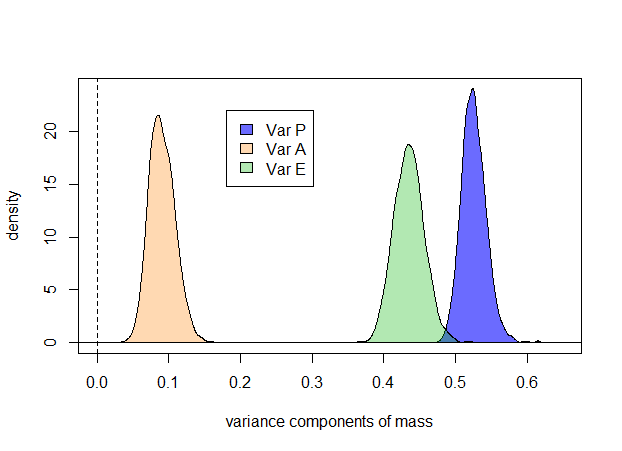


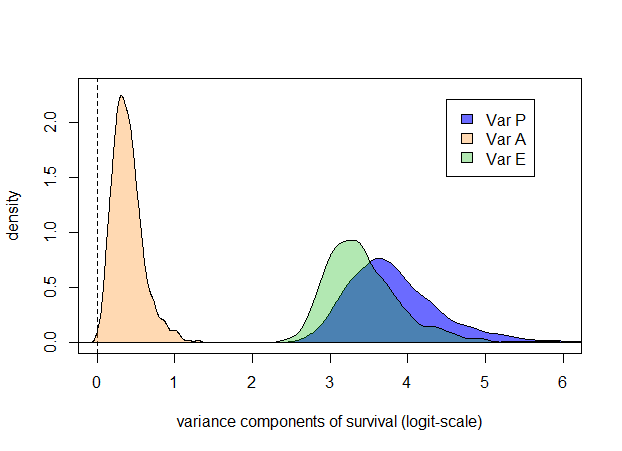


*Figure S1. Posterior distributions of the genetic and non-genetic variance components for nestling mass (top panel) and survival (bottom panel). ‘Var P’ represents total phenotypic variance, ‘Var A’ is additive genetic variance, and ‘Var E’ is the sum of all the other variance components. Based on 2000 samples from the posterior distribution of the MCMCglmm model shown in Table 1 (model I). Note that estimates for survival are on the latent scale.*

## Figure S2


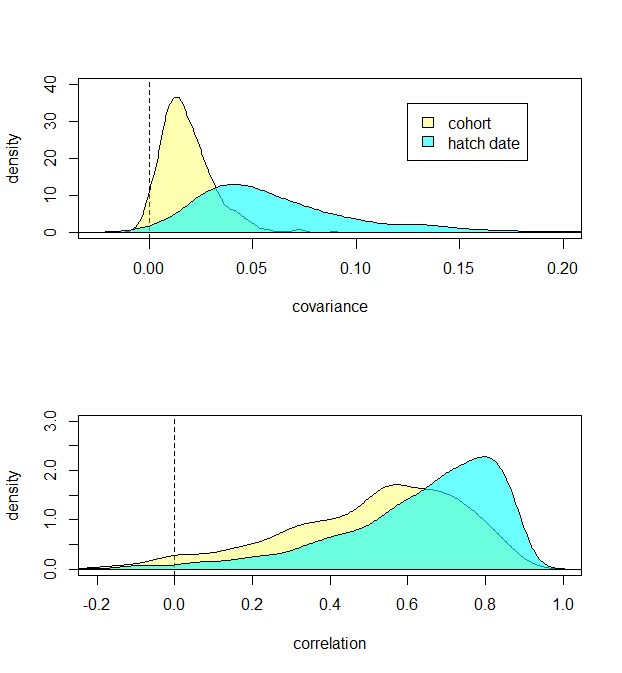


*Figure S2. Posterior distributions of the estimates of covariances (top) and correlations (bottom) between cohort (yellow) and hatch-date (blue) random effects, to show the difference in skew of the distributions. Based on 2000 samples from posterior distribution of the MCMCglmm model shown in Table 1.*

## Supplementary References

[1] Morrissey, M. B., Wilson, A. J., Pemberton, J. M. & Ferguson, M. M. 2007 A framework for power and sensitivity analyses for quantitative genetic studies of natural populations, and case studies in Soay sheep (*Ovis aries*). *J. Evolutionary Biology* **20**, 2309-2321.
